# Red Blood Cells and Tranexamic Acid in the Inhibition of Fibrinolysis

**DOI:** 10.1055/a-2609-3677

**Published:** 2025-06-03

**Authors:** Nikolett Wohner, Kata Balog Virag, Alexandra Raska, Krasimir Kolev

**Affiliations:** 1 Department of Biochemistry and Molecular Biology, Semmelweis University, Budapest, Hungary; 2 Department of Biochemistry, HCEMM-SU Thrombosis and Hemostasis Research Group, Semmelweis University, Budapest, Hungary

**Keywords:** tranexamic acid, red blood cells, fibrinolysis, synergism, postpartum hemorrhage

## Abstract

Tranexamic acid (TXA) is a widely used and cost-effective antifibrinolytic agent that has been utilized for decades in various clinical situations involving bleeding complications. Over time, a growing amount of clinical evidence has emerged, offering a robust basis for its applications.

However, in certain clinical scenarios, TXA appears to be less effective, highlighting the need for further research into its mechanisms of action beyond its well-known antifibrinolytic effects. One area of particular interest is the interaction between TXA and blood cells, especially red blood cells (RBCs). This interaction has garnered significant attention due to intriguing findings from large clinical trials, such as the WOMAN-1 and -2 studies, which evaluated the efficacy of TXA in preventing bleeding in postpartum hemorrhage, a major childbirth complication. These trials revealed that TXA was less effective in anemic patients, raising important questions about its broader mechanisms of action.

Emerging evidence suggests that the relationship between TXA and blood cells plays a crucial role at various stages of hemostasis, expanding its established role in fibrinolysis inhibition. Recent investigations into the interactions between TXA and RBCs have been particularly compelling and may provide valuable insights for improving clinical outcomes.

This review provides a fresh look at the interplay between RBCs, TXA, and the hemostatic system, focusing on basic molecular mechanisms and their potential implications for clinical practice.

Tranexamic acid (TXA) is a synthetic antifibrinolytic agent, a lysine analog that is widely used to manage bleeding in various clinical scenarios. Its primary mechanism of action involves reversible binding to plasminogen and its displacement from fibrin preventing its activation to plasmin by the fibrin-bound tissue-type plasminogen activator (tPA). Additionally, TXA exerts a direct inhibitory effect on the degradation of fibrin by plasmin. 1

Since its development in the 1960s, TXA has been utilized in the management of conditions characterized by excessive bleeding, including trauma, surgical procedures, hemophilia, and postpartum hemorrhage (PPH). 2 3

The safety, efficacy, and affordability of TXA have been demonstrated in numerous large-scale clinical trials. For instance, the CRASH-2 trial established the benefit of TXA in trauma patients by significantly reducing the risk of death due to bleeding when administered within 3 hours of injury. 4 Similarly, the WOMAN trial highlighted its utility in PPH, emphasizing its potential to save lives in low-resource settings. 5 Despite its established antifibrinolytic role, emerging data suggest that TXA's interactions with blood cells, particularly RBCs, may play a broader role in hemostasis, which warrants further exploration. 6

Recent studies have raised concerns about the reduced efficacy of TXA in anemic patient population. In response to this observation, the WOMAN-2 clinical trial was initiated to evaluate the efficacy of TXA in preventing PPH in anemic women. 7 Less understood aspects of TXA's mechanism of action in this context were further explored, focusing on its interactions with RBCs. A fundamental scientific study was conducted and published, highlighting a synergistic effect between RBCs and TXA in inhibiting fibrinolysis. 6 A better understanding of these mechanisms could lead to optimized clinical applications and improved treatment outcomes for bleeding-related disorders.

This review aims to provide an overview of the current knowledge on TXA, focusing on its multifaceted interactions with RBCs and their implications for hemostatic regulation.

## Red Blood Cells as Hemostatic Players

RBCs play an active role in hemostasis and are not merely passive bystanders. Several mechanisms have been identified through which RBCs contribute to thrombus formation. Clinical evidence indicates that increasing RBC counts can effectively manage certain bleeding disorders, even in cases where platelet levels remain unchanged or are reduced. 8 This suggests a complex and significant interplay between RBCs and other components of the coagulation system.

RBCs play a key role in determining blood viscosity, which rises with increasing hematocrit levels and serves as a contributing factor to thrombosis. 9 Elevated viscosity slows blood flow and acts as a potent prothrombotic element, aligning with Virchow's triad—a framework describing the causes of thrombosis as damaged vascular integrity, systemic hypercoagulability, and impaired blood flow. Higher viscosity can enhance platelet margination and influence how platelets interact with vessel walls, as platelet adhesion tends to increase with hematocrit levels. 10 Consequently, the effects of RBCs on hemostasis and thrombosis are influenced by both hematocrit levels and blood flow dynamics. Below a hematocrit level of 40% (V/V), the clot-stabilizing effect of RBCs decreases, and the bleeding tendency becomes clinically significant. 11 12 The movement of RBCs toward the center of the vessel also leads to a reduction in shear stress at the periphery, which decreases nitric oxide (NO) production. This, in turn, results in increased activation of platelets and endothelial cells. 13

RBCs display both prothrombotic and antifibrinolytic effects, which are driven by multiple mechanisms. RBCs promote thrombin generation by exposing phosphatidylserine, releasing prothrombotic microparticles, and exerting antifibrinolytic effects by structurally modifying the fibrin network. These changes influence plasminogen activation and alter the fibrin-cleaving activity of plasmin. 11 14 15

RBCs exert an antifibrinolytic effect due to their influence on fibrin structure, which alters plasminogen activation and the cleavage of fibrin by plasmin.

This antifibrinolytic effect, observed at physiologically relevant RBC counts, shifts the balance between coagulation and fibrinolysis toward clot formation and may help prevent the premature dissolution of hemostatic plugs.

The presence of RBCs leads to the formation of thinner fibrin fibers. This effect is mediated through an integrin receptor, such as eptifibatide—a blocker of the RBC fibrinogen receptor αIIbβ3—which not only reverses the impact of RBCs on fiber structure but also counteracts the RBC-related inhibition of fibrinolysis. 11

The effect of RBCs on fibrinolysis is also regulated by FXIII, which not only facilitates the covalent crosslinking of fibrin fibers but also mediates the retention of RBCs in contracting clots and determines the size of venous thrombi. This role of FXIII is not due to the direct crosslinking of RBCs to fibrin but rather the crosslinking of fibrin α-chains. 16

Clot contraction not only depends on platelets but also on fibrin, which serves as a critical connector between platelets, allowing the transmission of mechanical forces. Platelets contain actin and non-muscle myosin IIA, both of which are essential for the contraction process. Although it was once debated whether platelet–platelet interactions alone were sufficient, it is now widely accepted that fibrin is necessary for effective clot contraction.

One notable consequence of clot contraction is the deformation of RBCs into polyhedral shapes, known as polyhedrocytes. Polyhedrocyte formation plays a critical role in clot stability and mechanical resilience. Polyhedrocytes adopt a polyhedral shape due to tight packing in contracted blood clots. This structural transformation enhances clot density, reducing permeability and preventing premature clot degradation. These structures fail to form when platelet concentrations are low, likely due to insufficient force generation. If fibrinogen levels are low, higher platelet counts are required to form polyhedrocytes, possibly because some platelets cannot pull effectively without enough fibrin(ogen) to bind to. 17

In summary, RBCs significantly influence fibrinolysis by actively contributing to the formation of clot structure. Their absence leads to clots that are unstable and easily broken down, making anemia a potential risk factor for bleeding complications in certain clinical scenarios.

## Tranexamic Acid as an Antifibrinolytic Agent

TXA is a synthetic analog of lysine that blocks the lysine binding sites of plasmin(ogen), preventing its interaction with fibrin. Thereby, both the fibrin-dependent plasminogen activation by tPA and the fibrin degradation by plasmin are inhibited, preserving the integrity of the fibrin matrix created by the coagulation cascade and halting fibrin degradation. Compared to ε-aminocaproic acid, another lysine derivative, TXA is favored due to its significantly higher potency (about 10 times stronger inhibition of fibrinolysis on a molar basis) and longer plasma half-life. 18 It is available for oral, intravenous, and topical use. 19

TXA effectively inhibits fibrinolysis; hence it is widely used to reduce or prevent excessive bleeding in various medical and surgical settings. With a history spanning over 60 years, TXA has seen renewed attention in recent years following the release of several robust studies, particularly in severe trauma care and obstetrics. 2 5 20

The intravenous form of TXA has a half-life of approximately 30 minutes; however, studies in healthy individuals have shown that after 2 hours, plasma levels remain sufficient to achieve 80% inhibition of fibrinolysis. 21

Food intake does not influence the time to reach maximum concentration. The oral bioavailability of TXA is estimated at 33 to 34%. Its elimination follows an exponential pattern, with about 90% of the drug excreted in the urine within 24 hours. Renal clearance is the primary pathway for excretion, meaning patients with renal dysfunction are at an increased risk of complications. Consequently, dosage adjustments for both oral and intravenous forms should be based on serum creatinine levels. 22

As previously mentioned, compared to similar agents such as ε-aminocaproic acid, TXA demonstrates a significantly higher binding affinity for plasminogen and plasmin. This increased potency enables TXA to effectively suppress fibrinolysis, as indicated by lower serum D-dimer levels, while not affecting other serum markers of coagulopathy.

Furthermore, TXA's activity remains consistent even when administered alongside heparin. TXA and heparin affect different aspects of hemostasis, enabling TXA to stabilize clots and reduce bleeding even in anticoagulated patients. This makes it a valuable adjunct therapy in situations where anticoagulation is necessary but bleeding risk needs to be managed, particularly when TXA is applied topically. 23 24

In the next two sections, we will present the effects of TXA, categorized into its therapeutic and prophylactic applications.

## The Therapeutic Value of Tranexamic Acid in the Treatment of Manifest Bleeding

TXA is effective in a wide range of bleeding complications in different clinical fields.

### Obstetrics

The primary obstetric indications for TXA are abnormal uterine bleeding and PPH. TXA can reduce uterine bleeding by approximately 50%, with an effectiveness comparable to that of oral contraceptives, which achieve this effect through hormonal regulation and endometrial stabilization. 25 PPH warranted large international clinical trials: PPH is a critical obstetric condition and remains among the leading causes of maternal deaths globally. Defined as a blood loss of 500 to 1,000 mL within 24 hours after childbirth, PPH affects around 6% of pregnancies. Due to its significant impact on maternal health, the use of TXA as an additional treatment for PPH has been extensively researched.

The groundbreaking WOMAN trial involved more than 20,000 women experiencing PPH, comparing the outcomes of those treated with TXA versus a placebo. The results showed a notable reduction in deaths caused by hemorrhage in the TXA group (1.5% compared to 1.9%, *P* = 0.045), with no significant increase in thrombotic events. 5 The WHO has acknowledged TXA as a life-saving medication for managing PPH, and the NATA consensus statement advises administering TXA (1 g intravenously) as early as possible within the first 3 hours after PPH begins (GRADE 1B). 26 27

### Trauma

The CRASH-2 trial investigated the effect of TXA administration within 3 hours of trauma. The trial randomized over 20,000 trauma patients to receive either TXA or placebo, demonstrating a significant reduction in mortality (4.9% vs. 5.7%, *p* = 0.0077) with TXA treatment. Notably, the trial strongly supports early administration, with the greatest benefit observed when TXA is given within the first hour, and efficacy declining when administered beyond 3 hours post-injury. Delayed administration may result in established fibrinolysis, potentially explaining both the loss of benefit and the observed increased risk of death. Once plasminogen binds to fibrin, its conformation changes, enhancing its activation to plasmin. TXA's ability to displace fibrin-bound plasminogen is limited, making it less effective at inhibiting fibrinolysis once plasminogen is already associated with fibrin. This underscores the importance of administering TXA early, before significant fibrin binding occurs. 4 28

Meta-analyses of clinical trials have also confirmed that the effectiveness of TXA decreases with treatment delay; however, the underlying mechanism remained unclear. 29 A recent study provides experimental evidence supporting the theory that delayed treatment can lead to α2-antiplasmin depletion due to ongoing fibrinolysis. While TXA effectively stabilizes fibrin, it paradoxically enhances uPA-mediated plasminogen activation and prevents plasmin inhibition by α2-antiplasmin in circulation. This dysregulated plasmin activity contributes to coagulopathy by degrading essential coagulation factors, including fibrinogen, factor V, factor VIII, and factor XIII. 30

The CRASH-3 randomized controlled trial focused on patients with traumatic brain injury, comparing the effects of TXA to a placebo. Although TXA did not significantly reduce the overall risk of death due to head injury, it showed a notable benefit in reducing head injury-related deaths in patients with mild-to-moderate brain injury, especially when administered early after the trauma. 31

### Gastrointestinal Bleeding

We need to mention the specific field of gastrointestinal bleeding for which TXA does not seem to be effective in the treatment of bleeding complications. The effect of TXA on gastrointestinal bleeding was assessed in the HALT-IT trial, conducted on patients with acute gastrointestinal bleeding. Surprisingly, TXA did not reduce mortality and was associated with a higher risk of blood clots, potentially due to the large dose used or delayed treatment. 32 However, a recent review and analysis of multiple studies involving patients with gastrointestinal bleeding suggested that TXA effectively reduced ongoing bleeding and mortality when compared to placebo. The authors concluded that TXA is beneficial for upper gastrointestinal bleeding when given early, though its role in lower gastrointestinal bleeding requires further investigation.

The discrepancies between the HALT-IT trial and meta-analyses may be attributed to differences in study design, patient populations, TXA dosing regimens, and timing of administration. The HALT-IT trial utilized a high-dose intravenous TXA regimen, which did not demonstrate a mortality benefit and was associated with increased adverse events. In contrast, some analyses suggest that lower dose or enteral TXA may be effective in reducing hemorrhage, though more evidence is required to establish its safety and efficacy. 33 34 35

## The Value of Tranexamic Acid in the Prophylaxis of Bleeding Complications

The prophylactic use of TXA remains a topic of debate. However, studies conducted within the framework of patient blood management programs have provided growing evidence supporting its effectiveness in reducing blood loss across various major surgical procedures, particularly in cardiac and orthopedic surgeries. 33 34 35 36 37

TXA has demonstrated effectiveness in the prevention of PPH, a major cause of maternal mortality often associated with uterine atony. Systematic reviews of randomized controlled trials have confirmed that TXA, when administered alongside uterotonic agents during vaginal or cesarean deliveries, significantly reduces postpartum blood loss, the incidence of PPH, and the need for blood transfusions. 38 Notably, prophylactic use of TXA during cesarean delivery has shown a favorable safety profile, with no significant increase in thromboembolic complications. 39 Clinical consensus guidelines recommend the intravenous administration of TXA for women at elevated risk of PPH undergoing cesarean delivery. 27

On the contrary, the TRAAP trial randomized over 4,000 women to TXA or placebo for the prevention of PPH, and it did not find a significant difference in the rate of bleeding. 22

A clinical trial investigating the prophylactic use of TXA in thrombocytopenic patients with hematologic malignancies found no significant reduction in moderate-to-severe bleeding compared to placebo. 40 The study involved patients undergoing chemotherapy or stem cell transplantation, with TXA administered orally or intravenously for up to 30 days. There were no notable differences between the TXA and placebo groups in platelet transfusion needs, bleeding-free days, or thrombotic events. Common side effects, including diarrhea, fatigue, and nausea, were similar in both groups. Overall, TXA did not provide significant benefits for preventing bleeding in this patient population. 40 Further research is needed to clarify the pathomechanism underlying the findings of this large clinical trial. However, in addition to the impact of thrombocytopenia and the resulting altered hemostatic balance, an important factor to consider is the prophylactic use of TXA in this patient population, where its mechanism of action may differ from its role in treating active bleeding.

The WOMAN-2 trial examined the impact of TXA on PPH in women with moderate or severe anemia who delivered vaginally. The study concluded that administering TXA within 15 minutes of umbilical cord clamping or cutting did not significantly reduce the risk of clinically diagnosed PPH. 7

## Safety Aspects of Tranexamic Acid Usage

The primary concern limiting the use of TXA is the potential risk of thrombosis, although a recent meta-analysis found no significant increase in thrombosis associated with TXA. 41 Other possible adverse effects are rare but still important, with seizures being the most notable. For instance, in coronary artery bypass graft surgery, the higher incidence of seizures led to a reduction in TXA dosage. 42 However, seizures have been uncommon at the doses typically used in other clinical contexts.

## Interplay Between Tranexamic Acid and Red Blood Cells in the Stabilization of Fibrin

### Combined Effects on Fibrin Structure and Fibrinolysis

As discussed earlier, early TXA administration has been proven to reduce surgical blood loss and lower PPH-related mortality, particularly when given within 3 hours of birth. However, delayed treatment and the high prevalence of anemia among pregnant women further increase the risk and severity of PPH, highlighting the need for timely intervention. 5

As mentioned in the previous section, the WOMAN-2 trial specifically investigated the TXA effect on PPH in women with moderate or severe anemia after vaginal birth. Their findings revealed that administering TXA within 15 minutes of umbilical cord clamping did not significantly reduce the risk of clinically diagnosed PPH. Furthermore, the study found no evidence to suggest an increased incidence of thromboembolic events in these women. However, there was a notable increase in the risk of PPH among women who had experienced antepartum hemorrhage and received TXA. 7

Based on these clinical findings, fundamental research was undertaken to investigate the combined impact of TXA and RBC on fibrinolysis, determining whether their effects are merely additive or involve a synergistic interaction. 6 The study specifically examined how hematocrit levels and TXA concentrations jointly influence fibrinolysis inhibition. To explore their interaction, global fibrinolytic assays, including the ball sedimentation test and ClotPro thromboelastography, were utilized. Both tests measure all the phases of fibrinolysis—plasminogen activation by tPA within fibrin and the subsequent breakdown of fibrin by plasmin. The experiments demonstrated that the combined effect of RBC and TXA on fibrinolysis was stronger than the sum of their individual effects. This synergistic antifibrinolytic interaction was observed across clinically relevant hematocrit levels (up to 40% [V/V]). An alternative analysis using the parallel-line bioassay model, commonly employed in drug efficacy studies, further supported these findings. It showed a decrease in the inhibitory potency of TXA when hematocrit fell below 30% (V/V), particularly within the therapeutic TXA range exceeding 30 μM. 6

Scanning electron microscopy revealed contrasting effects on fibrin fiber size: RBCs produced thinner fibrin fibers, while TXA resulted in thicker fibers, particularly at higher RBC concentrations. 11 This observation aligns with the previously described role of lysine residues in fibrinogen in influencing fibrin polymerization. 6 Studies have shown that carbamylation of lysine residues on fibrinogen prevents their interaction with the corresponding lysine-binding sites on fibrin monomers, leading to fibrin clots with reduced fiber size and porosity. 43 44 As a lysine analog, TXA likely occupies these lysine-binding sites, mimicking the effects of lysine residues and thereby increasing fibrin fiber diameter. The diameter of fibrin fibers is inversely related to fiber density, branch points, and pore size within the fibrin matrix. RBC occupancy has been shown to significantly influence fibrinolysis rates. Thicker fibrin fibers are more susceptible to plasmin cleavage but less effective as templates for plasminogen activation by tPA. 45 Conversely, thinner fibrin fibers promote plasminogen activation but are more resistant to plasmin cleavage. 46 These differences in fibrin structure provide insights into the interplay between TXA, RBCs, and fibrinolysis.

The opposing changes in fibrin structure induced by TXA and RBC in this study suggest contrasting effects on the two stages of fibrinolysis—plasminogen activation by tPA and fibrin digestion by plasmin. TXA creates a fibrin structure that hinders plasminogen activation but promotes its degradation by plasmin. In contrast, RBCs produce a fibrin structure that enhances plasminogen activation but increases resistance to plasmin-mediated degradation.

Previous research has shown that TXA's inhibitory effect on plasminogen activation by tPA is significantly stronger than its effect on plasmin activity. 1 This suggests that inhibiting the initial stage of plasminogen activation is the key determinant in the overall fibrinolytic process. However, the opposing effects of RBCs and TXA on fibrin structure complicate predictions about their combined impact on plasminogen activation. Experimental evidence shows that TXA's ability to create thicker fibrin fibers, which are less effective templates for plasminogen activation, strongly influences the overall plasmin generation process, even when different ratios of RBCs and TXA are present. 6 Fibrinogen binds to RBCs via the αvβ3 integrin, affecting fibrin polymerization and the structure of the fibrin network. 47 Depending on the RBC-to-fibrin ratio, RBCs can connect varying numbers of fibrin strands, creating a template with different affinities and binding sites for plasminogen.

**Fig. 1:**
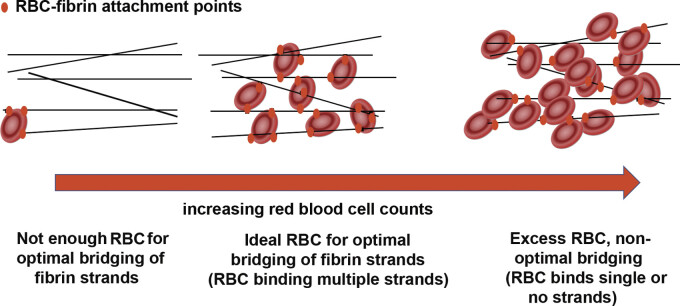
Effect of RBCs on fibrin stability and its response to tranexamic acid (TXA) inhibition. The presented scheme illustrates how RBCs interact with fibrin strands at different hematocrit values. At an intermediate RBC level (approximately 30%), the fibrin network is most effectively reinforced by strong connections between strands (middle panel). When RBC levels are lower, these connections are insufficiently formed (left panel). Conversely, at higher RBC concentrations, too many RBCs compete for the same limited binding sites on fibrin, weakening the network's stability (right panel). The figure is reproduced from Raska et al 6 published under CC-BY-NC-ND license ( http://creativecommons.org/licenses/by-nc-nd/4.0/ ).

At an optimal balance of RBC and fibrin, RBCs form bridges between fibrin strands, stabilizing the fibrin structure and limiting plasminogen binding. This allows TXA to more effectively block the lysine-binding sites of free plasminogen. However, at non-optimal RBC levels—whether too low or too high—the stabilizing effect diminishes. This can lead to more plasminogen binding to fibrin, reducing TXA's efficiency in inhibiting fibrinolysis ( Fig. 1 ).

The structural strength of fibrin, which serves as a biomechanical barrier, is crucial in determining how effectively TXA and RBC inhibit fibrinolysis. At optimal RBC/fibrin ratios, the fibrin structure is mechanically stabilized by RBC-formed bridges, leading to a stronger inhibitory effect. When these ratios deviate from the optimal level, this stabilizing effect is lost, resulting in reduced synergy between TXA and RBC. This bell-shaped response was evident in a global fibrinolysis assay where fibrin's structural integrity was tested by its ability to support a steel ball (ball sedimentation assay). 6

Experimental data from a model of traumatic liver injury in iron-deficient anemic mice demonstrate that pre-traumatic anemia significantly modifies the effects of periprocedural administration of TXA on bleeding outcomes and survival rates. 48 In nonanemic mice, TXA effectively reduced excessive bleeding, corrected trauma-induced coagulopathy, and inhibited fibrinolysis.

In contrast, the survival rate in anemic mice was significantly lower (approximately 50%) compared to nonanemic mice. Anemic mice exhibited more severe bleeding and fibrinolysis following trauma. Notably, although TXA abolished excessive bleeding, corrected the trauma-induced coagulopathy, restored the increased thrombin generation, and improved survival to approximately 80%—a rate comparable to that of nonanemic mice—it failed to inhibit fibrinolysis in the presence of anemia. This excessive fibrinolysis, characterized by elevated plasmin–antiplasmin complexes and D-dimers, developed independently of trauma-induced coagulopathy or bleeding. These pre-clinical observations underscore the critical role of anemia in trauma-related hemostatic dysregulation. Although TXA effectively manages certain aspects of coagulopathy in both anemic and nonanemic conditions, its inability to suppress fibrinolysis in anemic mice highlights the need for additional strategies to address the unique challenges posed by anemia in trauma patients. 48

### Effect of Factor XIII (FXIII) on the Interaction Between TXA and RBC

**Fig. 2:**
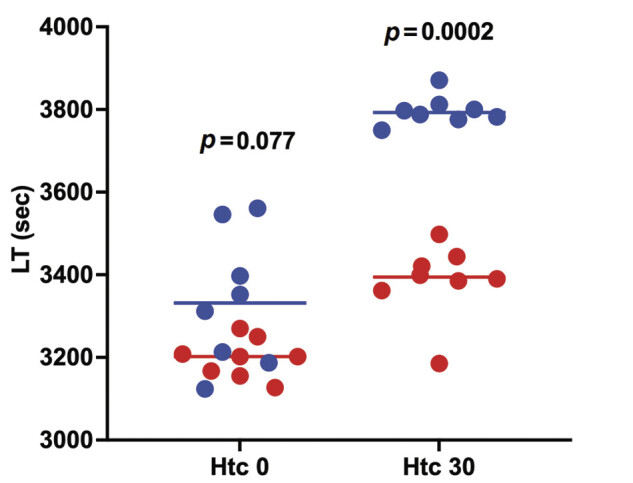
FXIII dependence of the combined antifibrinolytic effects of tranexamic acid (TXA) and RBCs. In ClotPro thromboelastography experiments performed as described in Raska et al, 6 fibrin clots were prepared from fibrinogen at 2 mg/mL, supplemented with 12 μg/mL plasminogen and 30% (v/v) RBCs, along with 128 μM TXA. Either recombinant FXIII at 10 μg/mL or FXIII inhibitor (2 μM) was added before fibrinogen was clotted with 8 IU/mL thrombin. The lysis time (LT), a parameter automatically provided by the ClotPro system (Enicor GmbH), was measured. LT shows time elapsed from the clotting time point until it reaches 50% of maximal clot firmness. Blue symbols show LT in the presence of FXIII while red symbols refer to the absence of FXIII. Statistical analysis was performed with Mann-Whitney U test ( *n* = 8). At a hematocrit (Htc) of 0, the extension of LT in the presence of FXIII is not statistically significant (ns, p = 0.077). However, at a hematocrit of 30, the addition of FXIII leads to a significant prolongation of LT as shown in the figure.

FXIII plays a key role in forming covalent bonds between fibrin strands, stabilizing fibrin clots, and facilitating RBC retraction within clots, as previously discussed. Thereby, a role for FXIII can be hypothesized in the combined effects of TXA and RBCs on fibrinolysis. *In vitro* experimental observations show that FXIII enhances the antifibrinolytic effect of TXA, but only in the presence of RBCs. Without erythrocytes, FXIII does not increase the efficacy of TXA in inhibiting fibrinolysis ( Fig. 2 ).

These findings expand our understanding of the interaction between RBCs and TXA. The presented results suggest that FXIII plays a pivotal role in modulating the action of TXA in cellular environments by reinforcing its antifibrinolytic synergism with RBCs. This interaction highlights the intricate relationship between FXIII, TXA, and RBC in stabilizing fibrin clots and reducing fibrinolysis. One potential explanation for this effect is the significant structural alterations observed within the fibrin meshwork. FXIII appears to strengthen the fibrin structure, contributing to a more robust network capable of resisting fibrinolysis. These structural changes provide a suitable environment for TXA and RBCs to synergistically inhibit plasminogen activation by tPA and fibrin degradation. The enhanced retention of RBCs within clots is a key factor underlying this phenomenon. FXIII facilitates the covalent cross-linking of fibrin strands, which improves the mechanical stability of clots and ensures that RBCs are tightly integrated into the fibrin matrix. This integration likely enhances the overall stability of the clot and amplifies the antifibrinolytic effects of TXA.

## Conclusion

**Fig. 3:**
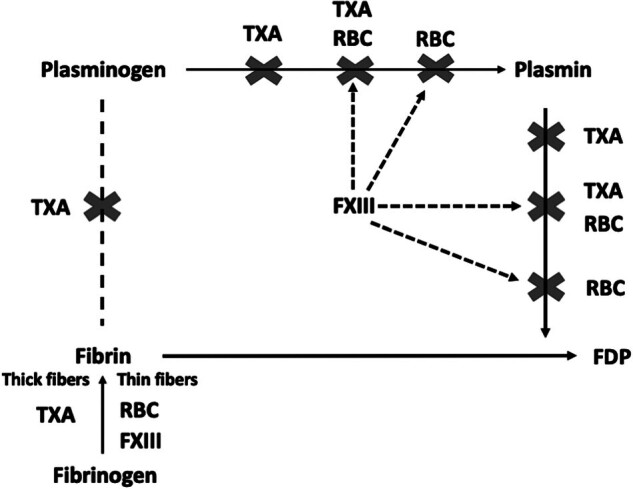
A schematic illustration depicting the key interactions between tranexamic acid, red blood cells, and factor XIII in the inhibition of fibrinolysis. Horizontal arrows indicate biochemical conversions catalyzed by enzymes represented by vertical arrows. Dashed lines illustrate binding interactions, while crisscross symbols indicate inhibitory effects. Dashed arrows represent the enhancement of inhibition. FDP, fibrin degradation products; FXIII, factor XIII; RBC, red blood cells; TXA, tranexamic acid.

The interaction between RBCs and TXA plays a crucial role in modulating fibrinolysis and stabilizing blood clots. Scientific evidence demonstrates that RBCs and TXA act synergistically to enhance their antifibrinolytic effect, with FXIII playing an integral role in this process. FXIII facilitates the cross-linking of fibrin strands, creating a stable clot matrix and promoting the retention of RBCs within clots, further reinforcing clot stability. This synergism, however, is significantly impacted by hematocrit levels. In anemia, where hematocrit is reduced, both the antifibrinolytic efficiency of TXA and the structural integrity of clots are compromised, leading to increased fibrinolysis, coagulopathy, and bleeding ( Fig. 3 ).

When TXA is administered in anemic conditions, its efficacy in suppressing fibrinolysis appears diminished, supporting the concept that the antifibrinolytic action of TXA is highly dependent on the presence of adequate RBC levels. These findings suggest that the correction of anemia is a crucial tool in managing bleeding complications, particularly in traumatic, surgical, or obstetric scenarios.

Additionally, the diminished efficacy of TXA in anemic conditions suggests that higher doses of TXA may be required to achieve the desired antifibrinolytic effect. While standard dosing of TXA has been optimized for nonanemic patients, anemic individuals may require tailored dosing regimens to account for the reduced hematocrit and associated fibrinolytic challenges. If anemia correction is not feasible, as is often the case in developing countries, adjusting the dose of TXA according to the patient's actual hematocrit level may be an effective strategy. This approach is particularly advantageous given the global availability and low cost of TXA, making it a viable option in resource-limited settings.

These considerations could improve patient outcomes across various clinical disciplines, achieving effective hemostasis, and minimizing mortality related to bleeding complications.
